# Role of NMDAR plasticity in a computational model of synaptic memory

**DOI:** 10.1038/s41598-021-00516-y

**Published:** 2021-10-27

**Authors:** Ekaterina D. Gribkova, Rhanor Gillette

**Affiliations:** 1 Neuroscience Program, University of Illinois at Urbana-Champaign, Urbana, IL USA; 2 Coordinated Science Laboratory, University of Illinois at Urbana-Champaign, Urbana, IL USA; 3 Department of Molecular and Integrative Physiology, University of Illinois at Urbana-Champaign, Urbana, IL USA

**Keywords:** Computational models, Computational neuroscience, Learning algorithms, Synaptic plasticity, Learning and memory

## Abstract

A largely unexplored question in neuronal plasticity is whether synapses are capable of encoding and learning the *timing* of synaptic inputs. We address this question in a computational model of synaptic input time difference learning (SITDL), where N‐methyl‐d‐aspartate receptor (NMDAR) isoform expression in silent synapses is affected by time differences between glutamate and voltage signals. We suggest that differences between NMDARs’ glutamate and voltage gate conductances induce modifications of the synapse’s NMDAR isoform population, consequently changing the timing of synaptic response. NMDAR expression at individual synapses can encode the precise time difference between signals. Thus, SITDL enables the learning and reconstruction of signals across multiple synapses of a single neuron. In addition to plausibly predicting the roles of NMDARs in synaptic plasticity, SITDL can be usefully applied in artificial neural network models.

## Introduction

Synaptic plasticity is a process in which synaptic properties are often modified, through activity-dependent changes in expression of post-synaptic receptors or in pre-synaptic neurotransmitter release. Most studies of synaptic plasticity in both physiological systems and artificial neural networks (ANNs) focus on the strengthening or weakening of synapses. Many of the studies emphasize NMDARs’ role in changing synaptic strengths and their importance in memory. However, only a few^1,2^ have examined another possible role where synaptic timing is changed, in terms of the actual speed of postsynaptic receptor activation. Here we propose that NMDARs are potentially important for changing synaptic timing in an activity-dependent manner, given that NMDAR dynamics can be regulated in many different ways, and NMDAR composition can change over time, with different subunits conferring distinct activation rates to NMDARs.

### NMDAR dynamics and expression

**Figure 1:**
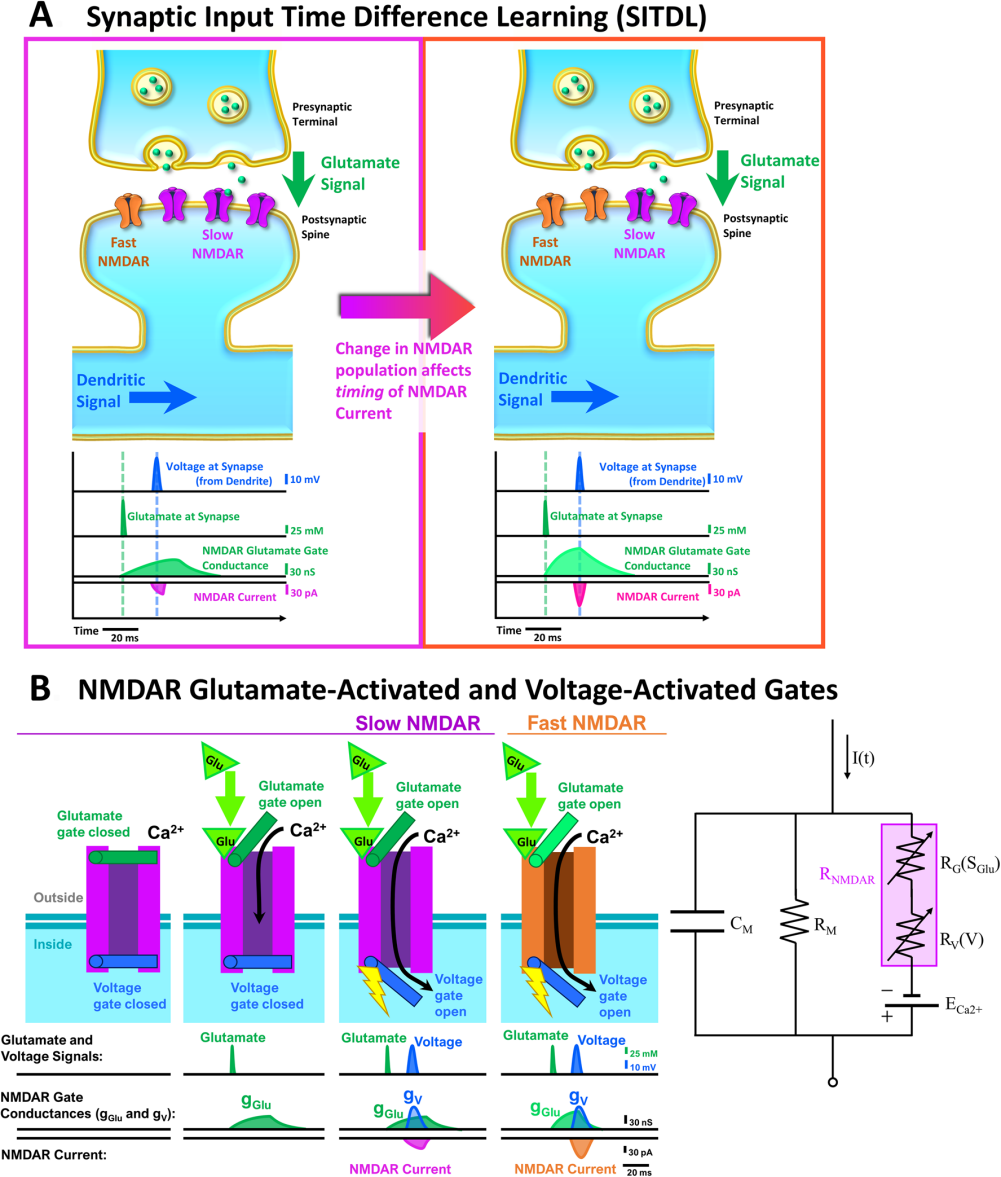
The SITDL hypothesis: An example of how activity-dependent changes in NMDAR population can affect synaptic current timing. (**A)** Left: A developing silent synapse starts with a majority of slow NMDARs with slower glutamate gate activation than fast NMDARs. The synapse receives a glutamate signal followed later by a dendritic voltage signal, causing a difference in activation of NMDARs’ voltage and glutamate gates. Right: As the synapse develops and experiences the same timing between voltage and glutamate signals, its NMDAR population may change, caused by the difference in glutamate and voltage gate activation. In this case, fast NMDARs replace slow NMDARs until the gate conductance difference is minimized, thus aligning the overall NMDAR current peak with the peak of the voltage signal. This allows the developing synapse to learn the timing difference between the voltage and glutamate signals by encoding it in the NMDAR population’s glutamate gate activation time. (**B**) Left: Each NMDAR has a glutamate gate and a voltage gate that can be activated independently, with corresponding gate conductances shown. Coincident activation of both gates allows influx of Ca^2+^ (NMDAR Current). A slow NMDAR has a slower glutamate gate activation, therefore experiencing slower change in glutamate gate conductance than a fast NMDAR. Right: Circuit diagram of a post-synaptic spine containing only NMDARs. An NMDAR (R_NMDAR_) is shown as variable serial resistances of its glutamate gate (R_G_) and voltage gate (R_V_) that depend on the glutamate signal (S_Glu_) and dendritic spine’s voltage (V), respectively. C_M_ denotes the membrane capacitance, R_M_ the membrane resistance, E_Ca2+_ the cell’s Nernst potential for Ca^2+^, and I(t) the external input.

NMDARs are heteromeric cation channels whose activation requires binding of glycine or D-serine, and glutamate, as well as local depolarization^3–6^. Essentially, the NMDAR acts as a coincidence detector, as it has both a glutamate gate and a voltage gate that require near-coincident activation for the NMDAR channel to fully open (Fig. 1). Note that throughout this paper, mentions of glutamate binding and activation of NMDAR’s glutamate gate imply coincident binding of the co-agonists glycine and D-serine. Once NMDARs are activated, they permit cation flow, including significant Ca^2+^ influx, to cause slow depolarization.

The dynamics of an NMDAR for glutamate binding and channel opening rates depend on its subunits. Each NMDAR is made of two obligatory GluN1 subunits, and two additional subunits of GluN2 or GluN3. Distinct GluN2 and GluN3 isoforms can determine NMDAR gating properties^7^. We focus on the GluN2 isoforms, GluN2A and GluN2B, as they predominate in many brain structures associated with memory and precise timing. GluN2A-containing NMDARs typically have faster activation, faster deactivation, and higher affinity for glutamate than GluN2B-containing NMDARs^7^. Notably, computational modeling of NMDAR subtype activation suggests that diheteromeric GluN1/GluN2A NMDARs have a rise time to peak activation of about 7 ms, while diheteromeric GluN1/GluN2B NMDARs have a markedly longer rise time of approximately 50 ms^8^. This suggests that changes in NMDAR subunit composition at a synapse effectively change how quickly glutamate activates the NMDAR glutamate gate, thus changing the timing of NMDAR-mediated Ca^2+^ influx.

There is significant regulation of NMDAR dynamics through ligand binding and post-translational modifications^4,9,10^. This includes a minimum of six distinct ligand binding sites on the NMDAR that can affect the probability of NMDAR channel opening, such as a polyamine regulatory site, and recognition sites for agonists and different ions. NMDARs also interact with proteins in the post-synaptic density. The anchoring of NMDARs to the post-synaptic density protein, PSD-95, through their GluN2 subunits, stabilizes their expression in the post-synaptic membrane and also couples them to intracellular signaling systems involving calmodulin^4^. The binding of the Ca^2+^-calmodulin complex to an NMDAR results in a Ca^2+^-dependent reduction of the NMDAR’s channel opening frequency and channel open time. Furthermore, NMDAR activity regulates casein kinase 2 phosphorylation of GluN2B subunits, which disrupts the subunits’ interactions with PSD-95 and decreases the GluN2B surface expression in neurons^9–11^. The wide diversity of ways in which NMDAR expression and dynamics can be modulated, through binding, post-translational modifications, and changing subunit composition, provides a wealth of opportunity for activity-dependent changes in synaptic current timing through NMDARs.

While NMDARs are present even at “silent” synapses, which do not generate detectable excitatory post-synaptic potentials (EPSPs) in response to neurotransmitter release^12^, specific NMDAR subunit expression varies over time and across brain regions^7^. For instance, GluN2B expression tends to be highest in early development, while GluN2A subunits are more widely expressed in adulthood. Notably, there is evidence of rapid activity-dependent bidirectional switching between GluN2A and GluN2B containing NMDARs, on the order of seconds in neonatal synapses^13^, and even in adult hippocampal synapses^14^. Distinct NMDAR expression is implicated not only in brain structures important for memory, but in other systems that rely on precise timing, such as in mammalian Calyx of Held and avian auditory brainstem^15–17^, important for inter-aural time and intensity difference calculations in sound localization, as well as in weakly electric fish relay cells, which are important for precise temporal regulation of the jamming avoidance response^18,19^. Notably, these delay line systems can resolve temporal disparities in the microsecond range, which may arise at the level of individual neurons. For instance, single neurons of the pre-pacemaker nucleus in a weakly electric fish are sensitive to temporal disparities as small as 1 µs, and their signaling and precision may involve NMDARs^20,21^. In one of the few studies on changes in NMDAR subunit composition during the development of a delay line system, it has been shown that GluN2A replace GluN2B subunits in chicken cochlear nuclei, after onset of hearing^17^.

### SITDL hypothesis

Much is still unknown about NMDARs for the intricacies of their dynamics and regulation, the roles of different subunits, and their specific roles in memory formation. Here, we propose a hypothetical model, Synaptic Input Time Difference Learning (SITDL), of how activity-dependent changes in fast and slow NMDAR expression could affect synaptic current timing. *In particular, we assume that synaptic NMDAR expression depends on differences in voltage and glutamate gate conductances, and that matching of these conductances provides optimal Ca*^2+^ *influx through NMDAR gates.* This process is driven by changes in subunit composition of the NMDARs, which determine glutamate gate characteristic activation time.

For example, the left panel of Fig. 1A shows a silent synapse having a majority of slow NMDARs, with slower activation times in response to glutamate than fast NMDARs. The slow NMDARs can resemble NMDARs containing GluN2B subunits, while fast NMDARs resemble those with GluN2A subunits. The silent synapse receives a synaptic glutamate signal followed by a voltage signal carried by the dendrite from a non-silent synapse, whose peaks occur several milliseconds apart. This difference in timing accompanies a difference in NMDAR voltage and glutamate gate conductance. We propose that as the synapse develops and experiences the same timing difference between voltage and glutamate signals, the difference in glutamate and voltage gate conductances causes its NMDAR population to change.

In the right panel of Fig. 1A, fast NMDARs replace slow NMDARs until there is a minimization of the difference in glutamate and voltage gate conductance, thus aligning the NMDAR glutamate gate conductance peak with the peak of the voltage signal. In effect, the synapse learns the timing difference between the voltage and glutamate signals, encoding it in the NMDAR population’s glutamate gate activation time, which is a function of the numbers of fast and slow NMDARs. Then, stabilization of a synaptic NMDAR population, such that there are no more removals and insertions of fast or slow NMDARs, possibly due to PSD-95 anchoring, would mean a stable memory of the timing difference. Note that silent synapses are particularly useful substrate for this SITDL mechanism, as glutamate signals alone do not cause depolarization of the synapse, thus providing a greater degree of independence between NMDAR glutamate gate and voltage gate activation.

Furthermore, maturation, which involves the insertion of α-amino-3-hydroxy-5-methyl-4-isoxazolepropionic receptors (AMPARs), may provide the synapse with a way of “recalling” the memorized timing difference. In this case, the NMDAR population no longer must rely on passing dendritic voltage signals for activation of their voltage gates, as AMPARs can provide depolarization in response to the glutamate signal. Thus, in theory, a glutamate signal alone could provide activation of NMDARs’ glutamate gates, and also indirectly provide depolarization to activate NMDARs’ voltage gates through the AMPARs. The resulting overall NMDAR current depends on the glutamate gate activation time of the stabilized NMDAR population, which encodes the previously memorized timing difference, in effect, recalling it.

In particular, SITDL and recall mechanisms may be useful for signal reconstruction when there are many silent redundant synapses and few non-silent synapses, as there often are in developing neurons^22^. Notably, networks of developing neurons have been shown to exhibit coordinated activity and rhythmic firing patterns^23,24^, essential to normal development of networks and synaptic connections. These developmental activity patterns are significantly different from those in mature circuits^25^. Thus, if a developing neuron receives rhythmic glutamate signals from presynaptic neurons, activation of a non-silent synapse would produce a rhythmic dendritic voltage signal in the neuron. Each of its silent synapses could therefore potentially receive both rhythmic voltage and glutamate signals, which would produce consistent overlap between signals. If these synapses could learn and encode the timing differences of these rhythmic signals during development, then once they mature, they would be able to recall and reproduce certain parts or patterns from the original developmental signals.

We explore the hypothesis of SITDL further and provide a computational single-compartment model (Methods). We also show that with many redundant silent synapses, as in a developing neuron, SITDL mechanisms, along with activity-dependent synaptic elimination and maturation, can enable reconstruction and recall of original developmental signals. Supplemental Slides S1 provide an overview and visualization of the SITDL mechanism and multi-synaptic signal reconstruction.

## Methods

### Input signals

To construct dendritic and glutamate signals to test the plasticity model, periodic input signals, S_N_ and S_Glu_ (Figure S2), were generated using a gaussian-like shape for the spikes in voltage, and a right-skewed shape for synaptic glutamate concentration^26–28^:

1 $$S_{N} = e^{{ - \,\,\left( {\frac{{(t - t_{N0} )^{2}}}{{\sigma_{N} }}} \right) }} ,$$

2 $$S_{Glu} = (t - t_{L0} )e^{{ - \alpha_{L} (t - t_{L0} )}} ,$$

3 $$I_{D} = I_{0} + S_{N} ,$$

where S_N_ = S_N_(t) determines the shape of the voltage input spike with parameters t_N0_ and σ_N._ Glutamate signal, S_Glu_ = S_Glu_(t) has characteristic right-skewed shape, with constant α_L_ that determines the width and decay of the spike, and t_L0_ is simply shifted relative to t_N0_ , with t_L0_ = t_N0_ − 1/α_L_, to ensure that the spike peaks of both S_N_(t) and S_Glu_(t) occur at the same exact time (Figure S2). The input signals, S_N_ and S_Glu_ were normalized by their respective maximums, S_NMax_ and S_GluMax_ such that $S_{N} = S_{N} /S_{NMax}$ and $S_{Glu} = S_{Glu} /S_{GluMax}$. The dendritic signal, I_D_ = I_D_ (t), depends on I_0_, a constant current, and the signal S_N_. Values for all constants are given in Table 1.

**Table 1:** SITDL model constants.

Symbol	Description	Values (no units, unless indicated)
σ_N_	Dispersion constant for S_N_	$0.0\overline{33}$
α_L_	Exponential constant for S_Glu_	10.0
I_0_	Constant current for I_D_	0.01
a_V_	Exponential constant for g_V_(t)	– 8.0
b_V_	Exponential constant for g_V_(t)	5.0
a_L_	Decay factor constant for g_L_(t)	0.999
b_L_	Signal input factor for g_L_(t)	0.065
Δt	Simulation time step constant	0.01 ms
τ_Fast_	Time constant for glutamate gate activation of fast NMDARs	7.0 ms
τ_Slow_	Time constant for glutamate gate activation of slow NMDARs	50.0 ms
n_Total_	Total number of NMDAR receptors	50 receptors
γ	Constant for scaling the time step Δτ	1.0
Δτ	Time step for changing τ_Glu_	0.05 ms
V_Rest_	Normalized resting potential	0.0
τ_R_	Time constant for V(t)	1.0 ms
k_D_	Constant for dendritic current contribution	3.9
k_S_	Constant for synaptic current contribution	0.40
τ_P_	Time constant for determining how slowly σ(t) changes	20.0 ms
Δτ_Max_	Maximum of Δτ_Glu_, used for modulating changes in σ(t)	0.0125 ms
a_P_	Exponential constant for logistic function of σ(t) in P(t)	0.30
b_P_	Exponential constant for logistic function of σ(t) in P(t)	– 70.0
δ	Threshold factor for synaptic elimination (for τ_D_ step = 8 ms and 2 ms, respectively)	1.002, and 1.0215

### NMDAR voltage and glutamate gate dynamics of a single synapse

NMDARs play important role in learning, memory, and development by regulating synaptic properties at the post-synaptic membrane. Each NMDAR is a heteromeric cation channel with a glutamate-activated gate and voltage-activated gate. Activations of the glutamate and voltage gates are largely independent, and either alone does not open the NMDAR channel completely. However, the activation of either gate will cause specific changes in conformation or channel structure and may potentially affect the NMDAR’s binding and phosphorylation sites. With near-coincident activation of both gates, the NMDAR channel opens, permitting Ca^2^^+^ influx.

We consider the glutamate and voltage gates as separate variable resistors, with each NMDAR composed of the glutamate gate resistor in series with the voltage gate resistor (Fig. 1B, Right). Therefore, in a single synapse, the overall post-synaptic NMDAR conductance, g = g(t), can be expressed as follows:

4 $$g = \frac{{g_{Glu} \cdot g_{V} }}{{g_{Glu} + g_{V} }},$$

where g_Glu_ = g_Glu_(t) represents the conductance of the NMDAR population’s glutamate gates, and g_V_ = g_V_(t) represents the conductance of its voltage gates. g determines the level of permeability and Ca^2+^ influx through channels of the NMDAR population (Fig. 1B, Left). The product of g_Glu_ and g_V_ in Eq. 4 also implies necessity of coincidence of glutamate and voltage gate activation.

The voltage dependence of an NMDAR has been previously modeled with experimental data as a logistic function of synaptic Mg^2+^ concentration and the post-synaptic voltage^29,30^. We can similarly describe the conductance of a population of NMDARs’ voltage gates, g_V_, as a simplified logistic function of the normalized voltage along the dendrite, V_D_ = V_D_ (t):

5 $$g_{V} = \frac{1}{{1 + e^{{a_{v} \cdot V_{D} + b_{v} }} }},$$

with constants a_v_ and b_v_ provided in Table 1. In Eq. 5, we assume that Mg^2+^ concentration stays relatively constant in the synapse, as expected for physiological conditions.

The evolution of g_Glu_ can be written as:

6 $$\frac{{dg_{Glu} }}{dt} = \frac{{g_{L} - g_{Glu} }}{{\tau_{Glu} }},$$

with approximate solution in the form of a single-time-step mapping:

7 $$g_{Glu}^{t + 1} = g_{L}^{t} + (g_{Glu}^{t} - g_{L}^{t} ) \cdot e^{{ - \Delta t/\tau_{Glu} }} ,$$

where

8 $$g_{L}^{t + 1} = a_{L} \cdot g_{L}^{t} + b_{L} \cdot S_{Glu} ,$$

τ_Glu_ is glutamate gate conductance characteristic time, Δt is a single time step, and a_L_ and b_L_ are constants between 0 and 1, the values for which are provided in Table 1. Here, we assume that glutamate gate conductance limit, g_L_, acts as an eligibility trace of glutamate signal S_Glu_ (Eq. 8), since NMDAR conductance depends on synaptic glutamate concentration and prior activations.

On a sub-second time scale, local changes in τ_Glu_ represent modification of receptors that transiently affect their dynamics and membrane stability, such as phosphorylation of NMDARs^9^. On a longer scale of seconds or more, overall changes in τ_Glu_ define the changes in numbers of slow and fast NMDARs, which significantly affect the dynamics of NMDAR glutamate gate conductance, g_Glu_. *A key point and assumption of the SITDL hypothesis is that NMDAR subunit composition, and consequent value of τ*_*Glu*_*, depend on the difference between the NMDAR gate conductances, g*_*Glu*_* and g*_*V*_*. So, the optimal Ca*^*2*+^ *influx occurs when the value of g*_*Glu*_* matches that of g*_*V*_. Following the assumption, temporal evolution of τ_Glu_ can be written as:

9 $$\tau_{Glu}^{t + 1} = \tau_{Glu}^{t} + \,\gamma \, \cdot \Delta \tau \cdot (g_{Glu} - g_{V} )(g_{L} - g_{Glu} ),$$

where Δτ is time step for τ_Glu_, γ is a scaling constant. The primary goal of the SITDL model is to find value of τ_Glu_ that minimizes the gate conductance mismatch, (g_Glu_ − g_V_), which would effectively represent the estimated time difference between the synaptic glutamate signal and the dendritic voltage signal.

Equation 9 results from gradient descent minimization of the function F(τ_Glu_) = (g_Glu_ − g_V_)^2^, with dF/dτ_Glu_ =− 2t(g_Glu_ − g_V_)(g_L_ − g_Glu_)/ τ_Glu_^2^. Equation 9 has two fixed points, at g_Glu_ = g_V_ and g_L_ = g_Glu_. Figure S4 shows evolution of (g_L_ − g_Glu_) and (g_Glu_ − g_V_) toward stable solution, where the peaks of g_Glu_ and g_V_ are aligned. Optimal solution is reached when g_Glu_ → g_V_ and g_L_ ≈ g_V_.

There are few studies modeling the dynamics of the NMDAR glutamate gate and how they change with different NMDAR subunit compositions. For instance, they primarily involve kinetic modeling of NMDARs with different subunit compositions^31–33^, but provide little suggestion for modeling the synaptic population of NMDARs, its overall dynamics, and how the NMDAR population may change over time in an activity-dependent manner. While τ_Glu_ describes the dynamics of NMDAR glutamate gate conductance, it depends on the glutamate signal and is difficult to compare to the experimentally known characteristics of slow and fast NMDARs. Therefore, it is useful to look at the NMDAR dynamics in response to a fixed signal: a single glutamate spike. For each synapse, we are particularly interested in the g_Glu_ rise-to-peak time, τ_Syn_, in response to a single glutamate spike, which is defined through the numbers of slow and fast NMDARs (n_Slow_ and n_Fast_)^34^:

10 $$\tau_{Syn} = \frac{{n_{Slow} \tau_{Slow} + n_{Fast} \tau_{Fast} }}{{n_{Total} }},$$

where n_Total_ is the total number of NMDAR receptors, τ_Fast_ and τ_Slow_ are known time constants of fast and slow NMDARs, respectively.

Using Eq. 14, and the assumption that n_Total_ stays constant, numbers of slow and fast NMDARs can be calculated from τ_Syn_ as follows:

11 $$n_{Slow} = \frac{{n_{Total} \left( {\tau_{Syn} - \tau_{Fast} } \right)}}{{\tau_{Slow} - \tau_{Fast} }},$$

12 $$n_{Fast} = n_{Total} - n_{Slow} .$$

τ_Syn_ is calculated as the rise-to-peak time of NMDAR glutamate gate conductance, g_Glu_, for a single glutamate spike as signal S_Glu_, similarly to previous studies^34^. τ_Syn_ dependence of τ_Glu_ is shown in Figure S3. τ_Syn_ values were calculated from g_Glu_ response to a single glutamate spike, at fixed τ_Glu_ vaues (ranging from τ_Glu_ = 1 ms to 2000 ms).

### Synaptic current and dendritic voltage

We can calculate the synaptic current, I_Syn_ = I_Syn_ (t), that results from NMDAR activation and consequent Ca^2+^ influx, using overall NMDAR conductance, g, and dendritic voltage, V_D_:

13 $$I_{Syn} = g \cdot V_{D} .$$

Changes in dendritic voltage are described via a single-compartment conductance-based model:

14 $$C\frac{{dV_{D} }}{dt} = \frac{{V_{Rest} - V_{D} }}{R} + k_{D} \cdot I_{D} (t - \tau_{D} ) + k_{S} \cdot I_{Syn} (t),$$

where V_Rest_ is resting membrane potential, τ_R_ = CR is a time constant, k_D_ and k_S_ are current contribution constants, τ_D_ is the dendritic delay time constant, indicating the time difference between synaptic and dendritic signal, I_D_(t − τ_D_) is delayed dendritic signal, and I_Syn_ is synaptic current resulting from NMDAR activation.

### Stabilization of glutamate gate conductance characteristic time τ_Glu_

The change in τ_Glu_ described by Eq. 9 is always dominated by the gate conductance mismatch. Therefore, unless there is a consistent and perfect match of the gate conductances, g_Glu_ and g_V_, τ_Glu_ will constantly change. We propose the existence of a stabilization mechanism that depends on the overall NMDAR conductance, g, such that when g is consistently large, τ_Glu_ will eventually stabilize. We modify Eq. 9 to include a stabilization variable, P, with the following set of equations:

15 $$\tau_{Glu}^{t + 1} = \tau_{Glu}^{t} + \,P \cdot \Delta \tau_{Glu} ,$$

where

16 $$\Delta \tau_{Glu} = \gamma \, \cdot \Delta \tau \cdot (g_{Glu} - g_{V} )(g_{L} - g_{Glu} ),$$

17 $$P = \frac{1}{{1 + e^{{a_{P} \cdot \sigma^{t} + b_{P} }} }},$$

18 $$\sigma^{t + 1} = \sigma^{t} + \frac{{g \cdot \left( {\Delta \tau_{Max} - |\Delta \tau_{Glu} |} \right)}}{{\tau_{P} }}.$$

P and σ define the stabilization mechanism: when g is consistently greater than zero (Eqs. 17, 18), and concurrently the change in τ_Glu_, Δτ_Glu_, is close to zero, τ_Glu_ stops changing. Δτ_Max_ is the maximum possible value for Δτ_Glu_. Its value and the values for constants a_P_, b_P_, and τ_P_ are provided in Table 1.

### SITDL multi-synaptic memory and recall

To test whether SITDL mechanisms could be used to memorize peak times of a glutamate signal and reconstruct it, several alterations are made. Multiple synapses are used, with each synapse represented as a single-compartment SITDL model with stabilization mechanisms (Eqs. 1–18), with its own dendritic delay time constant, τ_D_, and initial glutamate gate characteristic time, τ_Glu_. A set of “Learning Phase” simulations starts with a full set of synapses (i = 1, …,N), with 49 τ_D_ values uniformly distributed between 4 and 100 ms, and initial τ_Glu_ = 20 ms. In these simulations, each synapse receives a periodic glutamate signal as described before, and a sparse voltage signal with one spike repeating over the same period as the glutamate signal (Figs. 5, S2 bottom). For each synapse *i*, g_Avg_, the overall NMDA conductance averaged over the last 0.1% of the simulation time (last 150,000 points), is calculated. If a synapse’s g_Avg_ is lower than a threshold factor, δ, times the value of g_Avg_ averaged across the entire set of synapses, such that $\, g_{Avg}^{i} { < }\frac{\delta }{N}\sum\limits_{j = 1}^{N} {g_{Avg}^{j} }$, then it is eliminated. This is similar to the performance-based synaptic elimination rule used in the Clusteron model^35^, and to minimal-value deletion algorithms^36^. All synapses that have not been eliminated are considered stabilized, with τ_Glu_ permanently fixed. We assume that stabilized synapses express AMPARs, which depolarize the synapse upon binding glutamate. Therefore, for “Recall Phase” simulations, each stabilized synapse receives a single glutamate spike coincident with a single voltage spike (Fig. 5C). Note that k_D_ and k_S_, the constants for dendritic and synaptic current contribution to voltage, respectively, are set to 2.0 and 3.0 for these simulations. The resulting synaptic voltage traces are shifted by their corresponding τ_D_ and summed over all stabilized synapses to give a “recall signal”, which is compared against the original glutamate signal used during Learning Phase simulations. A smaller set of 12 synapses was also used for SITDL learning and recall simulations, with τ_D_ values uniformly distributed between 8 and 96 ms, and initial τ_Glu_ = 20 ms. “No-SITDL” simulations use the same Learning Phase and Recall Phase simulations, except there are no SITDL mechanisms during the Learning Phase, such that there are no changes in τ_Glu_ for any synapse.

### Mutual information (MI) analysis

A mutual information (MI) estimator (Adaptive partition using Interspike intervals MI Estimator (AIMIE)), was used to compare recall signal and original glutamate signal^37^. Short signals were repeated with proper periodicity, such that each signal had at least 4000 spikes for more accurate MI estimation. A higher MI estimate for the two signals suggests that they have a greater dependency and higher degree of similarity. AIMIE has been shown to work well with spike time series with disparate firing rates and may provide more accurate estimates of MI than several other commonly used MI estimators. Details on AIMIE’s calculations and its use in information flow analysis of a spiking network are provided in previous work^37^.

All simulations of the SITDL model were programmed and run in Spyder 3.0.0, a Python 3.5 environment. SITDL data were analyzed using MATLAB R2016b and Excel 2016.

### Code availability

The Python code for SITDL is available at https://github.com/KatyaGribkova/SITDL.

## Results

### The SITDL mechanism increases overlap of NMDAR gate conductances

The core mechanism of the SITDL model is learning the timing difference between synaptic glutamate and dendritic voltage signals. This involves changes in numbers of fast and slow NMDARs, and consequent changes in NMDAR glutamate gate characteristic time, τ_Glu_, and τ_Syn_, the corresponding rise-to-peak time of the glutamate gate conductance in response to a single glutamate spike. Using periodic glutamate and voltage signals and computational single-compartment model described in Methods (Eqs. 1–14), we ran multiple simulations for a range of initial values of dendritic delay, τ_D_, and τ_Glu_. It may be noted that the relative timing delay between signals, τ_D_, can result from travelling along post-synaptic dendrites, as well as from travelling along the pre-synaptic axon. Conduction delays in certain systems, such as corticothalamic projections and some unmyelinated axons, can range from less than 2 ms to as long as 100 ms^38–40^, and in development the regulation of myelination can also significantly affect conduction delays^41^.

**Figure 2:**
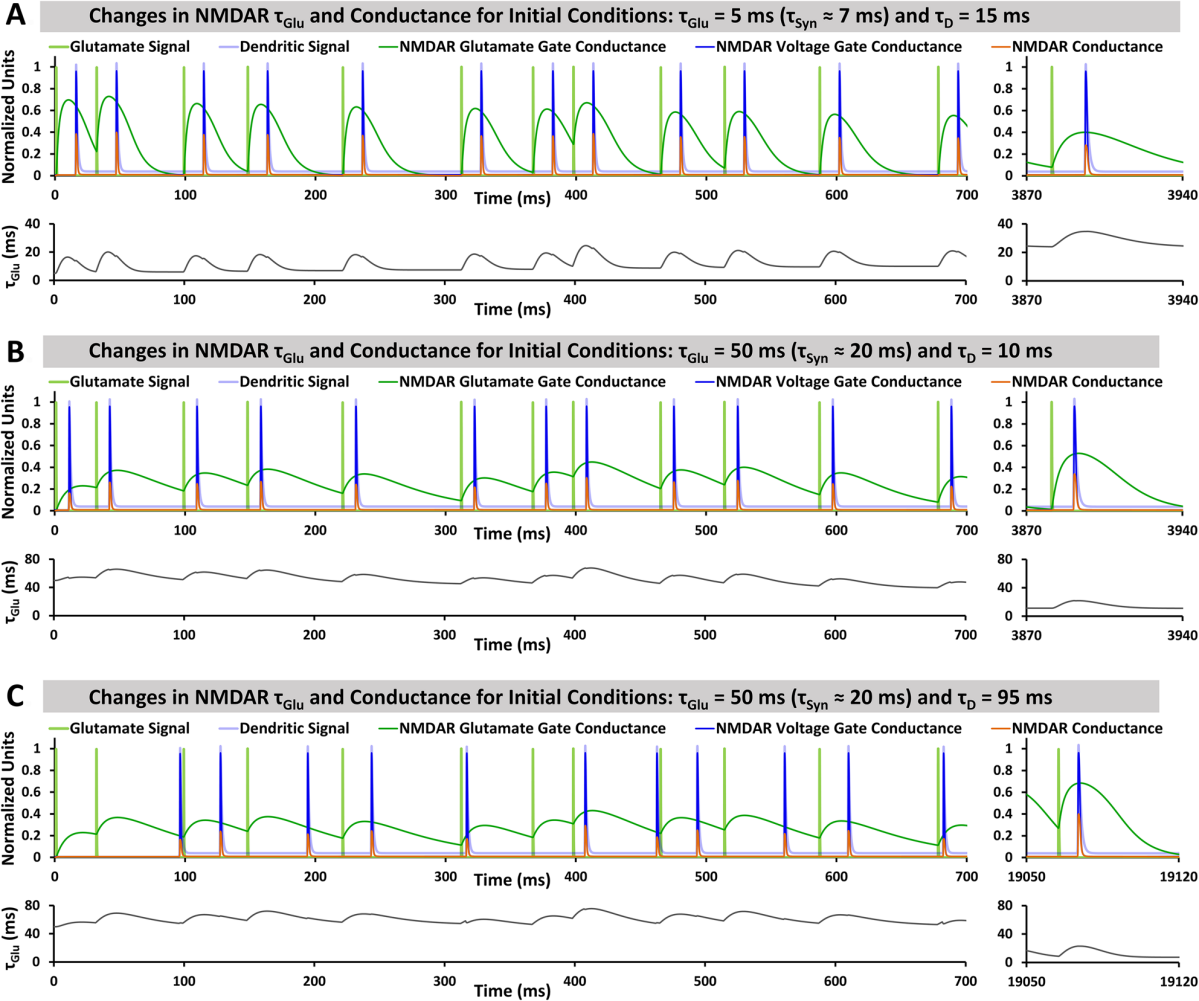
SITDL simulations of a single synapse receiving periodic inputs and showing evolution of τ_Glu_ and conductances for different initial conditions of τ_Glu_ and τ_D_. In each case, the synapse receives a synaptic glutamate signal (light green) followed by a dendritic voltage signal (light blue) delayed by time τ_D_. τ_Glu_ changes over time due to the NMDAR gate conductance mismatch. (**A)** Simulation with initial values τ_Glu_ = 5 ms and τ_D_ = 15 ms. Initially, glutamate gate conductance, g_Glu_, peaks before voltage gate conductance, g_V_. Over time, τ_Glu_ grows, delaying the g_Glu_ peak and achieving greater coincidence. (**B)** Simulation with initial values τ_Glu_ = 50 ms and τ_D_ = 10 ms. In this case, g_V_ initially peaks before g_Glu_. τ_Glu_ decreases over time, resulting in greater coincidence of the gate conductances. (**C)** Simulation with initial values τ_Glu_ = 50 ms and τ_D_ = 95 ms. Though there is much less overlap between glutamate and voltage signals, due to periodicity of the signal, the SITDL mechanism still appears to achieve greater coincidence over time.

Figure 2 shows the evolution of τ_Glu_ and NMDAR conductances for a single synapse receiving the glutamate signal, S_Glu_, followed by the dendritic voltage signal, V_D_. Both are copies of a similar periodic signal, with dendritic signal delayed by time τ_D_. The timing difference between signals produces a gate conductance mismatch, (g_Glu_ − g_V_) (Eq. 9), changing τ_Glu_ and shifting g_Glu_ peaks. This leads to greater overlap of the gate conductances. With initial values τ_Glu_ = 5 ms (corresponds to τ_Syn_ ≈ 7 ms) and τ_D_ = 15 ms (Fig. 2A), glutamate gate conductance, g_Glu_, initially peaks before voltage gate conductance, g_V_. This results in overall increase of τ_Glu_ over time. Coincidence appears to have been achieved at the end of the 4000 ms simulation. In Fig. 2B, g_V_ initially peaks before g_Glu_, and τ_Glu_ decreases over time, resulting in greater overlap of gate conductances. Figure 2C shows that SITDL mechanisms can still work to achieve greater gate conductance coincidence even when dendritic delay, τ_D_, is significant (τ_D_ = 95 ms) and there is much less overlap in signals.

**Figure 3:**
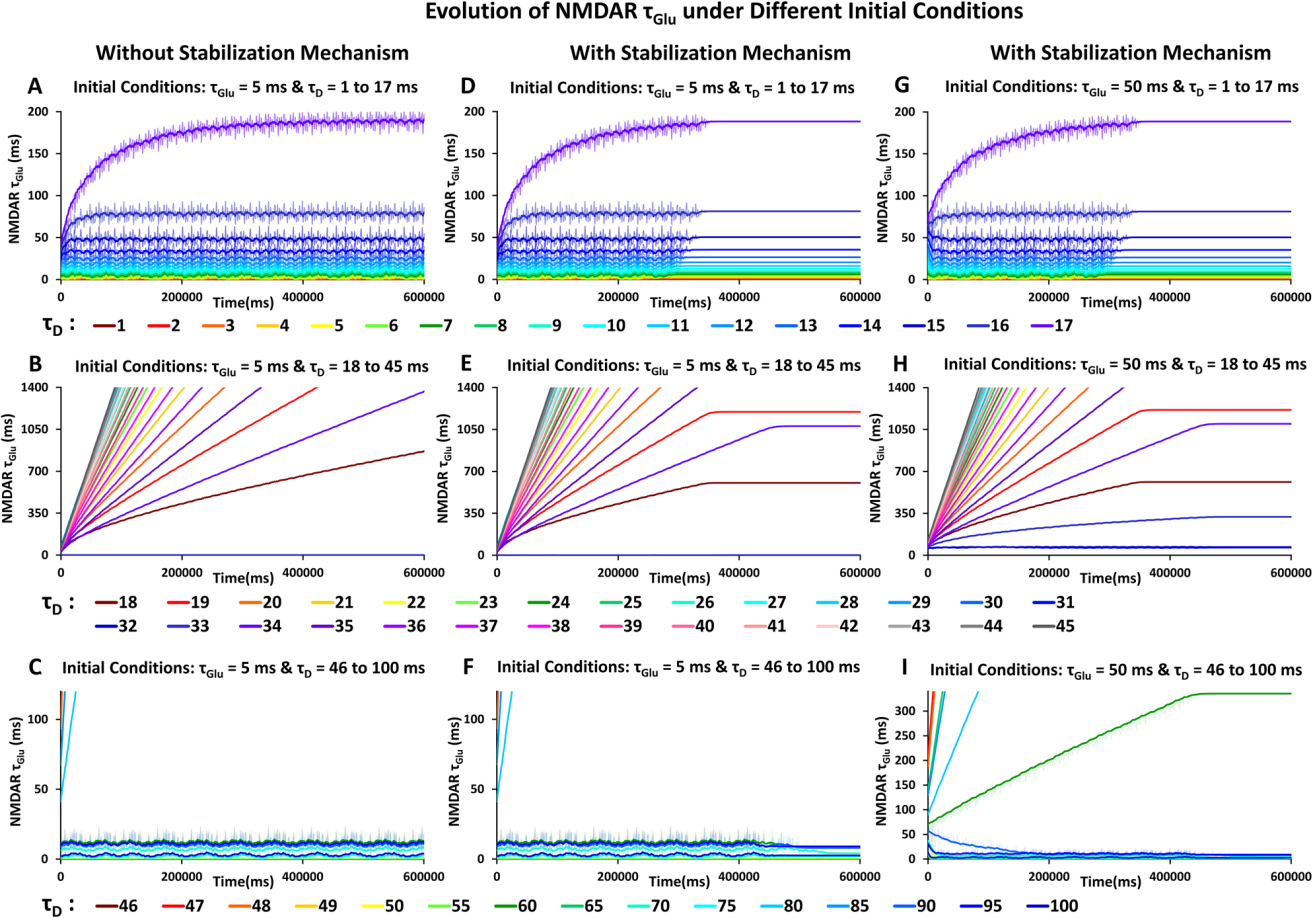
SITDL simulations with and without stabilization mechanisms, under different sets of initial conditions. Each curve shows the changes in τ_Glu_ over time, for a single simulation with the specific initial τ_D_ value indicated by the color of the curve. (**A)** Changes in τ_Glu_ when starting with τ_Glu_ = 5 ms and different initial τ_D_ value for each curve, ranging from 1 to 17 ms. By 600,000 ms simulation time, each curve appears to reach more stable values. (**B)** Changes in τ_Glu_ with initial conditions τ_Glu_ = 5 ms and τ_D_ value ranging from 18 to 45 ms. In most cases, τ_Glu_ increases, but there does not appear to be sufficient simulation time for it to reach more stable values as in (**A)**. (**C)** Changes in τ_Glu_ with initial conditions τ_Glu_ = 5 ms and τ_D_ value ranging from 46 to 100 ms, which provide much less overlap of glutamate and voltage signals. Certain τ_D_ values, such as 49 to 60 ms, 70 to 75 ms, and 90 to 100 ms, seem to provide sufficient overlap for τ_Glu_ to change significantly and potentially achieve coincidence of gate conductances with enough simulation time. (**D–F)** Same simulations and initial conditions as in Fig. 4A–C, but with stabilization mechanisms, which cause τ_Glu_ to stop changing when there is sufficient overall NMDAR conductance. Note, τ_Glu_ stabilization generally occurs more quickly for smaller τ_D_, and even at high τ_D_ (18 to 100 ms), τ_Glu_ still stabilizes for certain values at the end of the simulation, despite limited overlap of the periodic signals. (**G–I)** Same simulations with stability mechanisms as in Figure (**D**–**F**), except with initial τ_Glu_ = 50 ms.

Figure 3A–C shows simulations of the SITDL model with a greater range of initial delays and longer simulation time of 600,000 ms to explore the evolution of τ_Glu_. For initial τ_Glu_ = 5 ms, and τ_D_ = 1 ms to 17 ms, τ_Glu_ seems to quickly attain relatively stable values, with little change towards the end of the simulation. The smaller the τ_D_, the more quickly τ_Glu_ stabilizes. This can be explained by smaller gate conductance mismatch and greater overlap between glutamate and voltage signals. For τ_D_ = 18 to 45 ms (Fig. 3B), τ_Glu_ grows but does not stabilize as much as for smaller τ_D_. For some τ_D_, τ_Glu_ keeps growing past 1000 ms without achieving greater stability, suggesting an insufficient overlap of signals. Likewise, for τ_D_ = 46 to 100 ms (Fig. 3C), only certain τ_D_ values, such as τ_D_ = 49 to 60 ms, 70 to 75 ms, and 90 to 100 ms, seem to provide sufficient overlap for τ_Glu_ to approach small stable values at the end of the 600,000 ms simulation time. Different initial τ_Glu_ and longer simulation times may allow τ_Glu_ to stabilize, paticularly for τ_D_ values larger than 17 ms, and to potentially achieve coincidence of glutamate and voltage gate conductances.

It may be noted that while τ_Glu_ approaches a more stable range of values in most cases, τ_Glu_ does not fully stabilize in Fig. 3A–C. Thus, we made key alterations to the SITDL model, using Eqs. 15–18 for implementing τ_Glu_ stabilization mechanisms, which are important for demonstrating stable memory formation and recall. Figure 3D–F demonstrates simulations of this variation of SITDL model for τ_D_ ranging from 1 to 100 ms, similar to Fig. 3A–C, with initial τ_Glu_ of 5 ms. Likewise, Fig. 3G–I shows that τ_Glu_ can decrease from a higher initial value (τ_Glu_ = 50 ms), and fully stabilize at values similar to those in Fig. 3D–F, due to SITDL stabilization mechanism. In some cases, the higher initial τ_Glu_ allows for stabilization at additional τ_D_ values (Fig. 3H). While we showed that τ_Glu_ is able to stabilize at values lower than 5 ms (Fig. 3A,D,G) to demonstrate the range of capabilities of the SITDL algorithm, for all subsequent simulations we limited τ_Glu_ to values between 5 and 1410 ms to correspond to the more biologically relevant NMDAR conductance rise-to-peak time limits of around 7 ms and 50 ms, respectively.

### NMDAR τ_Glu_ corresponds to fractions of slow and fast NMDARs

We assumed that τ_Glu_ is determined by the numbers of fast and slow NMDARs. Fast NMDARs can be considered those with GluN1/GluN2A subunit composition, with estimated rise-to-peak time, τ_Syn_, of 7 ms, and slow NMDARs as those with GluN1/GluN2B subunit composition, with estimated rise-to-peak time τ_Syn_ of 50 ms^8^. Over the course of development, slow NMDARs are typically replaced with fast NMDARs^7^. As a test, we ran a SITDL model simulation with initial values τ_Glu_ = 150 ms and τ_D_ = 10 ms, and calculated numbers of fast and slow NMDARs using estimated τ_Syn_ and Eqs. 10–12 with n_Total_ = 50, τ_Fast_ = 7 ms, and τ_Slow_ = 50 ms. τ_Syn_ was estimated from τ_Glu_ values, using linear interpolation of data points from the τ_Syn_ vs. τ_Glu_ plot of Figure S3.

**Figure 4:**
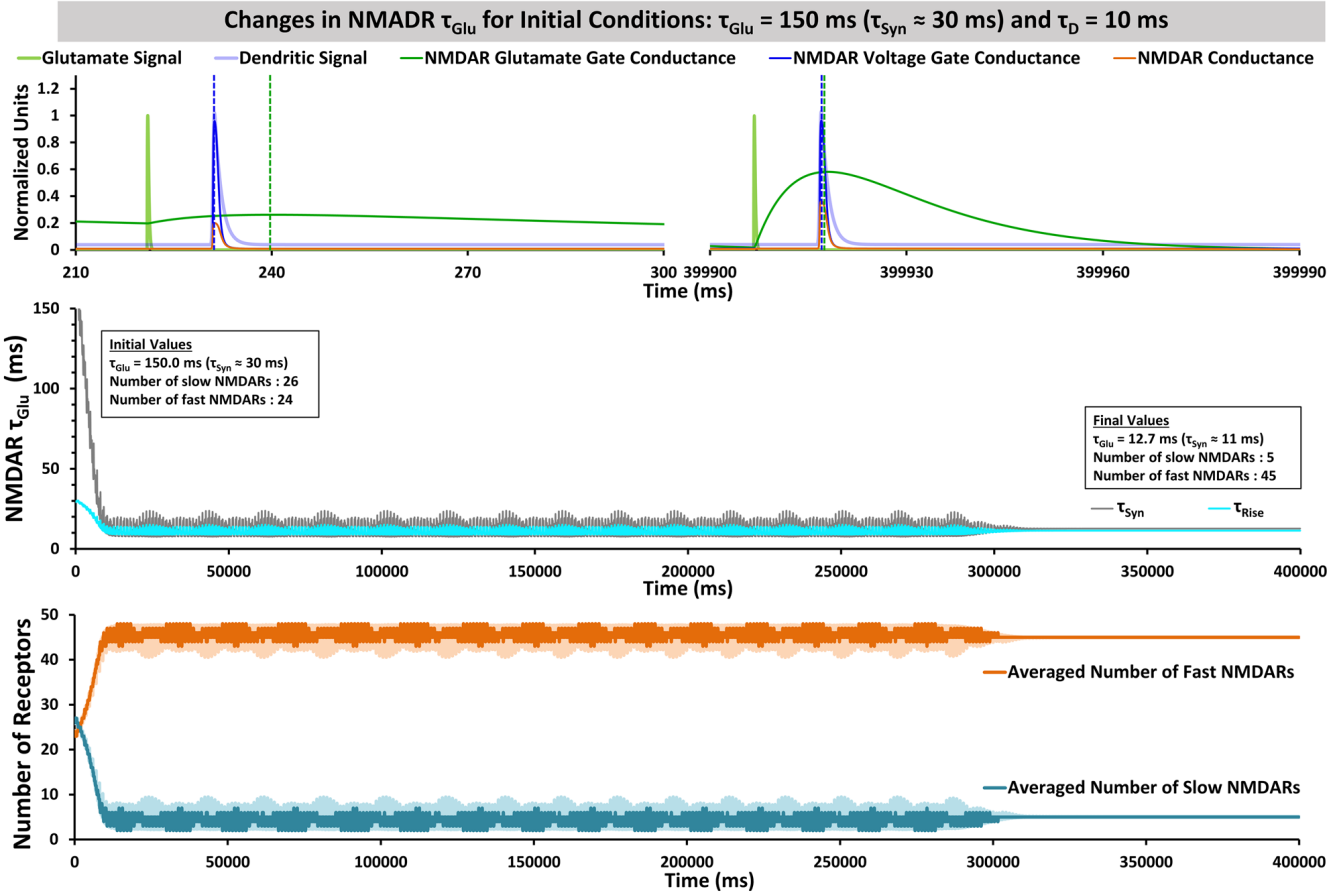
Simulation of the SITDL synapse model, showing changes in τ_Glu_, and corresponding changes in numbers of slow and fast NMDARs. This simulation was run with plasticity and stabilization mechanisms for τ_Glu_, with initial conditions τ_Glu_ = 150 ms and τ_D_ = 10 ms, and with constants for calculating the numbers of slow and fast NMDARs n_Total_ = 50, τ_Fast_ = 7 ms, and τ_Slow_ = 50 ms. (**A)** Segments of 90 ms close to the start and end of the simulation are shown at the left and right, respectively, with g_Glu_ and g_V_ peaks (green and blue dashed lines) noticeably becoming more coincident. (**B)** shows the time course of τ_Glu_, with an initial value of 150 ms (τ_Syn_ ≈ 30 ms), which corresponds to the synapse having 26 slow NMDARs and 24 fast NMDARs. τ_Glu_ decreases overall, with some oscillation, until it stabilizes at 12.7 ms (τ_Syn_ ≈ 11 ms), which corresponds to 5 slow NMDARs and 45 fast NMDARs in the synapse, indicating a replacement of slow NMDARs with fast NMDARs. (**C)** shows the corresponding numbers of slow and fast NDMARs over time, each averaged over a 800 ms time window (darker traces).

Because τ_Glu_ decreases, the g_Glu_ maximum shifts significantly closer to g_V_ maximum (Fig. 4A). Initially, τ_Glu_ = 150 ms (τ_Syn_ ≈ 30 ms), with 26 slow NMDARs and 24 fast NMDARs. As τ_Glu_ decreases and stabilizes at its final value of ~ 12.7 ms (τ_Syn_ ≈ 11 ms), there are 5 slow NMDARs and 45 fast NMDARs (Fig. 4B,C). Reduction of τ_Glu_ can therefore represent a replacement of slow NMDARs with fast NMDARs, and τ_Glu_ convergence can be considered as stabilization in expression of fast and slow NMDARs.

### SITDL mechanisms across multiple synapses enable reconstruction of synaptic signal

**Figure 5:**
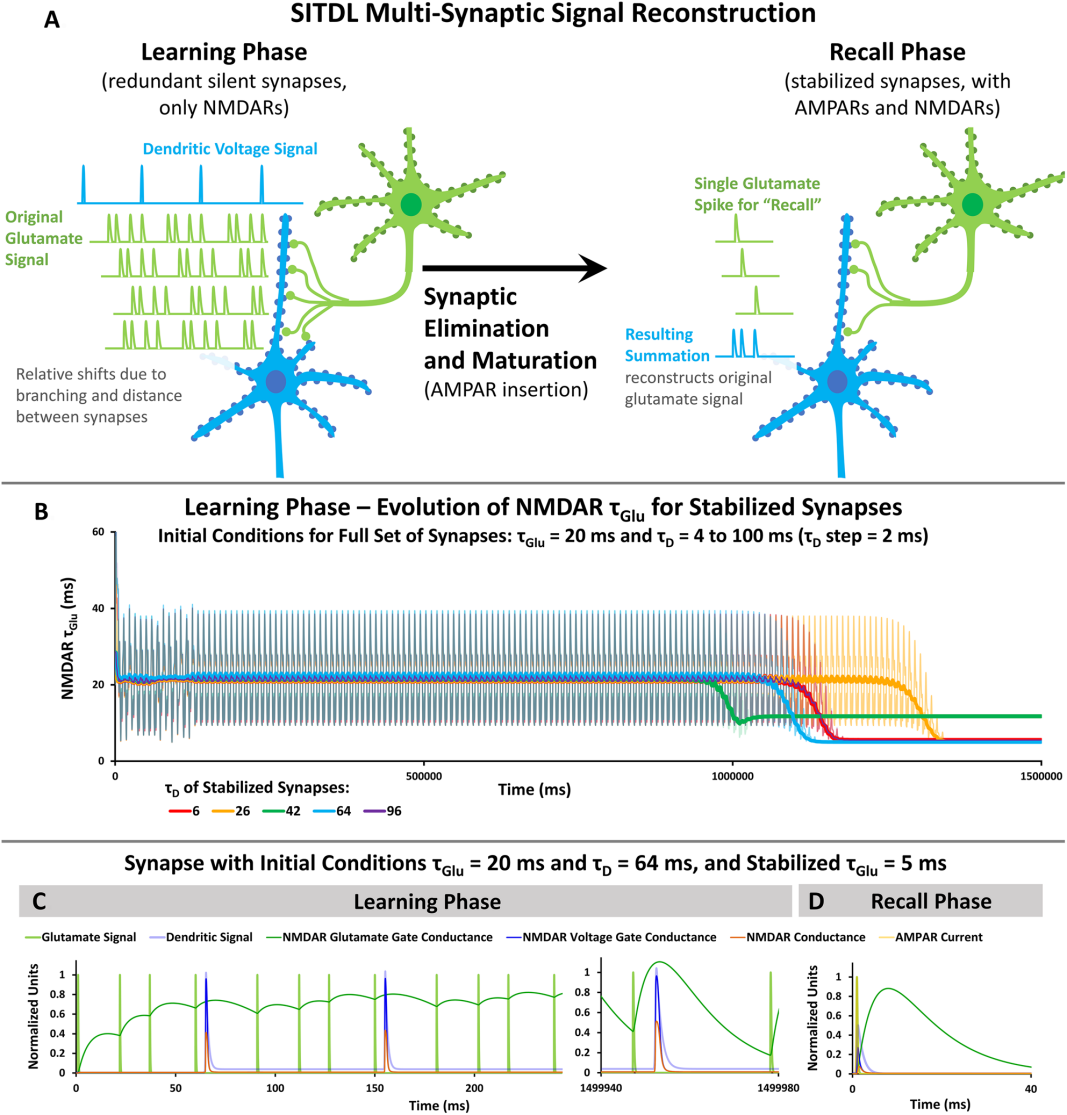
Multi-synaptic SITDL simulations of learning. (**A)** The Learning Phase starts with a large number of redundant synapses, each receiving the same periodic glutamate signal, delayed relative to a similarly periodic but sparse voltage signal by dendritic time constant τ_D_. After learning, synapses are eliminated or stabilized based on overall NMDAR conductance. During the Recall Phase each mature stabilized synapse receives a single glutamate spike. The summation of resulting voltages across all stabilized mature synapses with the appropriate dendritic delays, produces a reconstruction of the original glutamate signal received during the learning phase. (**B)** Evolution of τ_Glu_ for synapses that were stabilized at the end of Learning Phase simulations. The full set of synapses started with initial conditions τ_Glu_ = 20 ms and τ_D_ values uniformly distributed from 4 to 100 ms, with a step of 2 ms. Synapses with insufficient average overall NMDAR conductance were eliminated, leaving only stabilized synapses, whose corresponding τ_D_ are shown. (**C)** An example of a Learning Phase simulation, showing how NMDAR conductances change over time for a synapse with initial τ_Glu_ = 20 ms and τ_D_ = 64 ms. The synapse receives a periodic glutamate signal and a sparse voltage signal with the same period. The first voltage spike occurs close to the fourth spike of the glutamate signal. At the end of the simulation the average overall NMDAR conductance is sufficient for this synapse to be stabilized. (**D)** Recall Phase simulation of stabilized synapse from (**C)** with final values τ_Glu_ = 5.0 ms and τ_D_ = 64 ms. Since stabilized synapses are assumed to express AMPARS, in this case the synapse receives a single glutamate spike coincident with a single voltage spike.

The potential for SITDL mechanisms to achieve greater coincidence between NMDAR glutamate and voltage gate conductances, even with very limited overlap of signals, suggests that they could be used to memorize peak times of a glutamate signal and reconstruct it. To explore this, large numbers of SITDL synapses, with stabilization mechanisms and τ_D_ values uniformly distributed between 4 and 100 ms, were run through Learning Phase simulations like the previous simulations in Fig. 3, with ranges of different initial conditions. During the Learning Phase simulation, each synapse receives a periodic glutamate signal, and a sparse rhythmic voltage signal of the same period, delayed relative to the glutamate signal by time constant, τ_D_ (Fig. 5A,C). We can consider this initial set of synapses to be located along a neuron’s dendrite, receiving the same glutamate signal from the branched axon of a single pre-synaptic neuron (Fig. 5A). These types of redundant multi-synaptic connections are seen in neocortex, striatum and hippocampus^42–45^, and they may also arise as a result of long-term potentiation (LTP)^46^. Notably, this synaptic multiplicity appears to increase during development in hippocampal CA3-CA1 synapses^47,48^.

As before, τ_Glu_ and conductance values change over the course of the Learning Phase simulations (Fig. 5B,C). After simulations end, synapses with insufficient overall NMDAR conductance are eliminated, similar to NMDAR-dependent and Ca^2+^-based synaptic elimination in biological neurons^49,50^, and to synaptic elimination mechanisms in computational models such as the Clusteron^35^, and minimal-value deletion algorithms^36^. Figure 5B shows the changes in τ_Glu_ for synapses that were stabilized, with an initial set of synapses having initial conditions τ_Glu_ = 20 ms, and τ_D_ uniformly distributed from 4 to 100 ms, with a step size of 2 ms. The τ_D_ step can represent how far apart the initial synapses are on the dendrite (Fig. 5A). The leftover stabilized synapses are assumed to express AMPARs. These were run through Recall Phase simulations, where each stabilized synapse received a single glutamate spike and a single voltage spike that coincided due to AMPAR expression (Fig. 5D).

**Figure 6:**
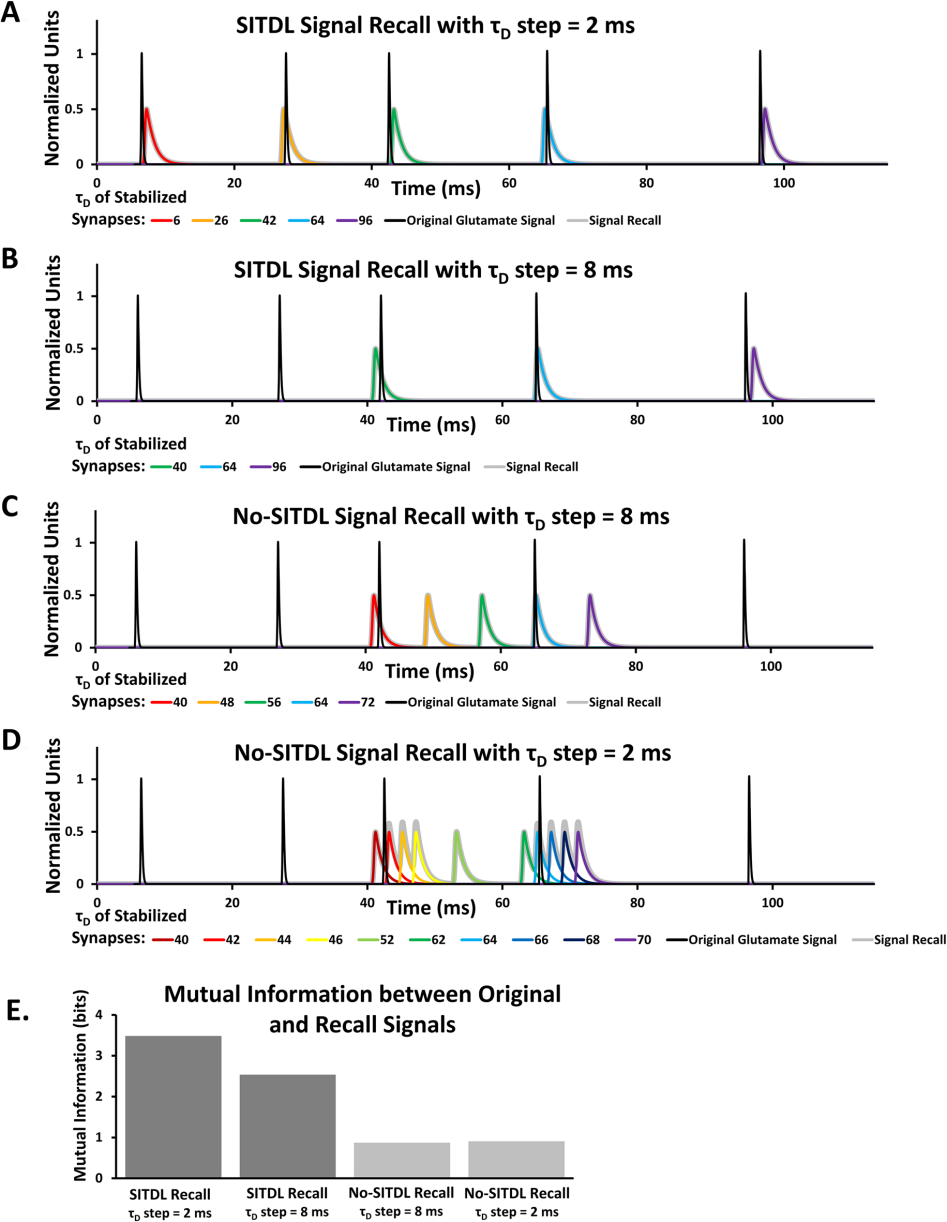
Results of Recall Phase simulations for synapses with and without SITDL mechanisms. There are 4 different sets of synaptic simulations, each with their own starting conditions: 2 sets of SITDL simulations with τ_D_ step = 2 ms and 8 ms, and 2 sets of simulations without SITDL mechanisms with τ_D_ step = 2 ms and 8 ms. For all simulation sets, τ_D_ values are initially distributed uniformly with the corresponding τ_D_ step, and all initial τ_Glu_ = 20 ms. For each simulation set, following Learning Phase simulations, synaptic elimination, and Recall Phase simulations, a recall signal is obtained by summating the resulting Recall Phase voltage signals (Fig. 5C), shifted by corresponding τ_D_ (**A**–**D** traces in color), over all stabilized synapses. Note, to provide better visual comparison of original and recall signals, the original signal was slightly shifted relative to the recall signal, with a delay of 5.5 ms for (**A,D**), and 5 ms for (**B**,**C**). (**A)** The recall signal for SITDL simulation set with τ_D_ step = 2 ms very closely resembles the original glutamate signal that was used during Learning Phase. (**B)** Recall signal for SITDL simulation set with τ_D_ step = 8 ms is less similar to the original glutamate signal, because of the much larger τ_D_ step used during Learning Phase. (**C,D)** Recall signals for no-SITDL simulation sets with τ_D_ step = 8 ms and 2 ms, respectively. There are no changes in τ_Glu_ in these simulations, thus synapses are unable to achieve greater overlap of glutamate and voltage gate conductances. Even with synapses that were stabilized, the peaks of the recall signal are significantly shifted from the peaks of the original glutamate signal. (**E)** Estimates of MI between original glutamate signal and recall signal for each simulation set, using MI estimator AIMIE^37^. SITDL simulation sets, particularly the one with smaller τ_D_ step (2 ms), provide greater MI estimates than no-SITDL simulation sets, suggesting greater degree of similarity between their reconstructed recall signal and the original glutamate signal.

The summation of resulting Recall Phase voltage signals across the stabilized synapses (Fig. 6, traces in color), shifted by the corresponding τ_D_ value, provides a “recall signal” that very closely resembles the original glutamate signal from the Learning Phase (Fig. 6A). For recall signals constructed from SITDL simulations with much larger τ_D_ step, there are less spikes and peak times are slightly less similar to those of the glutamate signal (Fig. 6B). Furthermore, recall signals constructed from simulation sets (Learning Phase, elimination, Recall Phase) without SITDL mechanisms such that τ_Glu_ cannot change, appear to be even less similar to the original glutamate signal, as they are missing the first, second, and fifth glutamate spikes, and have additional spikes in the vicinity of the third and fourth glutamate spikes (Fig. 6C,D). Using the MI estimator AIMIE^37^, we show that there is a greater degree of similarity between the original signal and recall signal reconstructed from SITDL simulations (Fig. 6E). SITDL mechanisms across a set of synapses, particularly when they are distributed closely enough, can therefore be used to effectively memorize and recall synaptic signals.

## Discussion

SITDL explores the potential for a single synapse to learn the timing of input signals through changes of its receptor dynamics. In the computationally simple SITDL model we show that single synapse can “learn” the timing difference between glutamate and dendritic voltage signals. *The functioning principle of single-compartment SITDL model is to reach optimal combination of fast and slow NMDAR subunits and corresponding glutamate gate conductance characteristic time, τ*_*Glu*_*, through minimization of NMDAR gate conductance mismatch, (g*_*Glu*_ − *g*_*V*_*).* τ_Glu_ stabilizes when optimal Ca^2+^ influx is achieved. Decreases in τ_Glu_ correspond to a replacement of slow NMDARs by fast NMDARs (Fig. 4), as is typical during neural development. However, depending on initial conditions, such as initial τ_Glu_ value and dendritic delay τ_D_, τ_Glu_ can also increase over time (Fig. 3), relating to the observed bidirectional switching of fast and slow NMDARs in hippocampus^13,51^.

NMDARs are particularly suited to mediate relations of the SITDL hypothesis because of their largely independent glutamate and voltage gates. SITDL mechanisms would likewise function under the assumption of NMDAR’s slow Mg^2+^ unblock^52^. Furthermore, it is possible that other receptors with coincidence detector properties, such as the inositol 1,4,5-trisphosphate receptor (IP3R)^53,54^, could be involved in SITDL-like mechanisms. Additionally, a variety of ligand-gated ion channels also have active voltage-sensitive conductances that could dispose them toward various ways of regulation in sensitive ranges of voltage. While the assumptions of the SITDL model have not yet been tested in physiological experiments, the functional principles may significantly expand synaptic capabilities in both biological and artificial systems.

Much is still unknown about NMDAR properties, and their activity-dependent modifications. It is possible that specific changes in NMDAR conformation, occurring independently for glutamate and voltage gate activation, can convey the mismatch between gate conductances through interfacing with cellular signaling pathways. For instance, if τ_Glu_ is too large, with too many slow NMDARs at a synapse, then glutamate gate activation peaks after the voltage gate activation, producing a specific combined conformational state in NMDARs, particularly slow ones, that indicates the NMDAR gate conductance mismatch. This specific conformational state could affect the NMDAR’s anchoring with post-synaptic density proteins, such as PSD-95. Therefore, NMDARs with the highest mismatch, as indicated by their specific combined conformational state, can be removed from the membrane, and replaced with other NMDARs. This NMDAR cycling process can continue until the balance of slow and fast receptors is found, where glutamate and voltage gate conductances peak at the same time, for an optimal τ_Glu_. The different nanodomain organization of slow and fast NMDARs in the postsynaptic density could also provide more dynamic cycling and replacement of NMDARs^55,56^, such that unstable slow NMDARs with high mismatch would be more likely replaced with fast NMDARs, and vice versa. Moreover, SITDL provides potential mechanisms for new learning rules in ANNs, which currently employ primarily synaptic weight changes.

The SITDL model can increase overlap of g_Glu_ and g_V_ in a synapse that receives very similar periodic signals with small relative time shifts (Fig. 2). This may be particularly useful for developing synapses in delay line systems like those of mammalian and avian auditory brainstem and sensory circuits of weakly electric fish^57,58^, all of which rely on very precise timing of signals. Some delay line systems resolve temporal disparities in the microsecond range even with just a single neuron^21^, and some depend on NMDARs in development^17^. Notably, there have been few computational models for achieving precise timing in delay line systems. One such model explores temporal precision in the barn owl auditory system^59^, using unsupervised Hebbian learning rules and a broad random distribution of transmission delays. While this may show how delay line systems with large numbers of neurons are able to achieve such temporal precision, it does not explain how it can be achieved with much more limited numbers of neurons and transmission delays, such as in pre-pacemaker nucleus neurons of weakly electric fish^21^. Furthermore, the vast diversity of neuronal morphology and circuit organization, as found in cerebral cortex and cerebellum^60,61^, may require fine-tuning of synaptic input timing. Even without τ_Glu_ stabilization mechanisms, the SITDL model can still increase overlap of NMDAR gate conductances with larger dendritic delays, such as for some τ_D_ greater than 45 ms, which provide very little overlap of glutamate and voltage signals (Figs. 2C and 3). Due to periodicity of the signals, less frequent but repeated overlap can cause significant changes in τ_Glu_. With τ_Glu_ stabilization mechanisms depending on overall changes in τ_Glu_, and overall NMDAR conductance, the SITDL model stabilizes τ_Glu_ even with quite small overlap between glutamate and voltage signals. Changes in τ_Glu_ are bidirectional (Fig. 3), and represent replacement of fast and slow NMDARs (Fig. 4). This relates to mechanisms of rapid bidirectional switching in NMDAR subunit compositions of developing hippocampus^13^.

Achieving greater gate conductance overlap with very limited overlap of signals may be quite useful, especially in memory formation. For instance, in a neuron that receives highly delayed copies of the same or similarly periodic signal, any memorized timing differences may represent the commonly occurring inter-spike intervals of the signal. We show in SITDL simulations that it is possible to memorize and reconstruct the original synaptic glutamate signal (Figs. 5, 6). Notably, synapses which stabilized at the smallest τ_Glu_ values provided the largest NMDAR conductances, saving them from synaptic elimination.

### Potential tests of SITDL in biological systems

SITDL mechanisms would be particularly valuable in systems that rely on precise timing and sequence memorization, such as the neural circuits involved in avian song and human speech learning^40,62^, as well as spatial map formation^63^. If we consider the auto-associative networks of the CA3 region of the hippocampus, where synapses can exhibit bidirectional changes in NMDAR subunit compositions^51^, then the SITDL mechanism could potentially be used for sequence memorization in single neurons. Figure S5 shows one potential mechanism of sequence learning in CA3, with each synapse learning the timing between two distinct inputs. Notably, in hippocampal neurons, the distribution and nanoscale organization of GluN2B subunits vary significantly between proximal and distal synapses^64^. Likewise, spike timing dependent plasticity (STDP) rules of synapses also vary in a location-dependent manner^65^. SITDL mechanisms could potentially be used to establish specific NMDAR expression, and consequently the synaptic timing required for the distinct location-dependent STDP rules, along the dendritic arbor.

To test for the existence of SITDL mechanisms in real neurons, in vitro studies of neuronal cultures and brain slices, such as those of hippocampus and developing neocortex could be useful. In particular, it would be interesting to observe axonal and synaptic activity across multiple points in developing multi-synaptically connected neurons, through multi-electrode recordings or with calcium and voltage indicators. Furthermore, neuronal activity might be manipulated, pharmacologically or with stimulating electrodes, alongside single particle tracking of different NMDAR subunits in post-synaptic membranes^66^, to provide more insight into possible existence of neuronal SITDL mechanisms. Molecular analyses of NMDARs in these cases, including conformational changes and phosphorylation site alterations under different stimulation protocols, might provide a deeper understanding of the potential mechanisms involved. For instance, it is possible that NMDAR gate conductance mismatch could be due to the different conformational changes caused by glutamate binding and depolarization of an NMDAR. These specific conformational changes could alter the NMDAR’s phosphorylation sites, modifying its dynamics and anchoring stability in the post-synaptic membrane, and leading to potential subunit switching.

## Supplementary Information

Supplementary Information.
