# Supplementary material for: Role of NMDAR plasticity in a computational model of synaptic memory
Source: Sci Rep. 2021 Oct 27;11:21182. doi: 10.1038/s41598-021-00516-y (PMC8551337; doi:10.1038/s41598-021-00516-y)
Supplement: Supplementary file 1 — Supplementary Information. [file 41598_2021_516_MOESM1_ESM.docx]

Supplementary Information for

**Role of NMDAR Plasticity in a Computational Model of Synaptic Memory**

Ekaterina D. Gribkova and Rhanor Gillette

Ekaterina D. Gribkova

Email: gribkov2@illinois.edu

**This file includes:**

Supplemental Slides S1

Figure S2

Figure S3

Figure S4

Figure S5

**Supplemental Slides S1 (embedded PowerPoint file, double-click slide above to show).** Synaptic Input Time Difference Learning (SITDL): Mechanisms across multiple synapses enable signal reconstruction. Slides provide an overview and visualization of SITDL mechanisms and how they can enable multi-synaptic signal reconstruction.


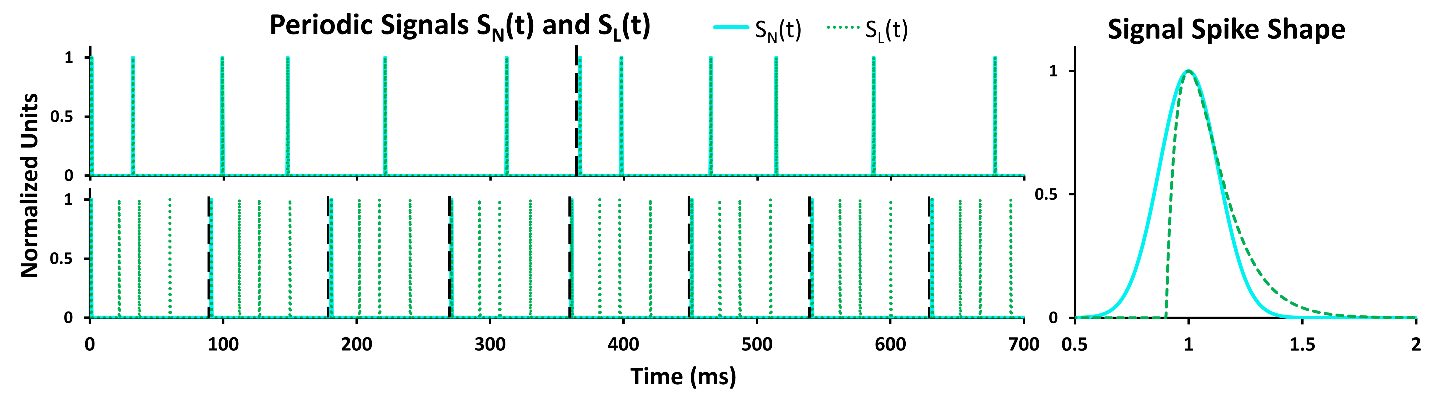


**Figure S2**. Periodic signals S_N_(t) and S_Glu_(t) used for voltage and glutamate signal generation, respectively. Left: Top plot shows signals used for single synapse SITDL simulations, while bottom plot shows signals used for multi-synaptic SITDL simulations. The timing of peaks follows a repeating pattern, as indicated by the vertical black dashed lines. Right: S_N_(t) spikes have a gaussian-like shape, while S_Glu_(t) spikes have a right-skewed shape.

**
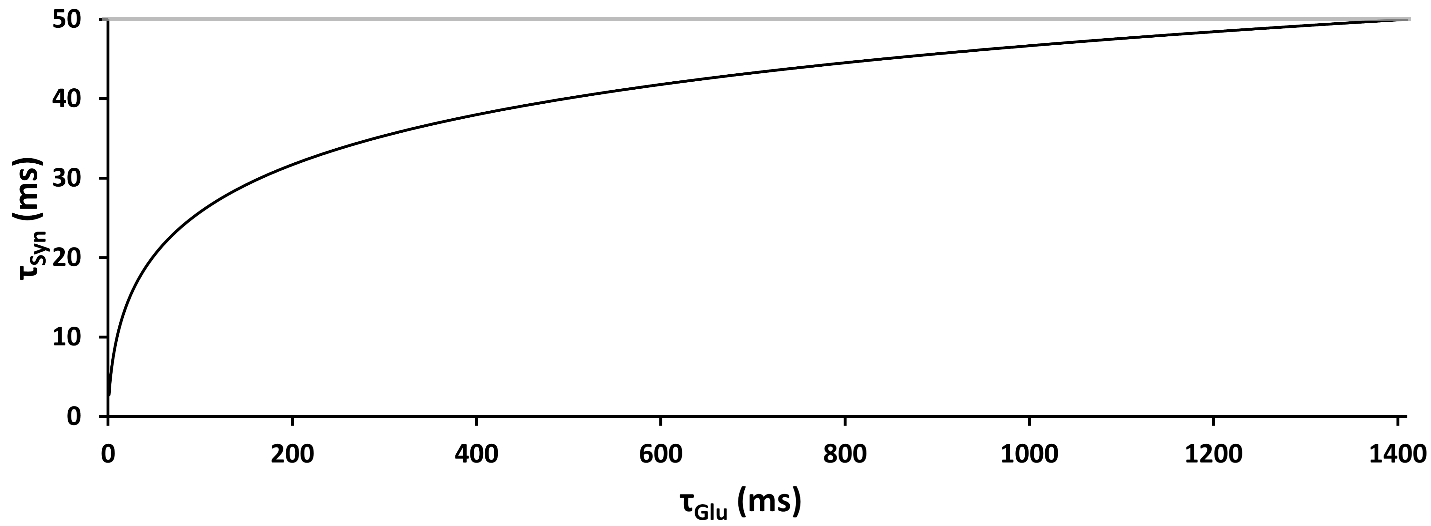
**

**Figure S3**. Graph comparing NMDAR glutamate gate activation time, τ_Glu_, against NMDAR glutamate conductance rise-to-peak time, τ_Syn_. Corresponding τ_Syn_ values were estimated for values of τ_Glu_ ranging from 1 ms to 1410 ms with 1 ms step, using SITDL simulations. Each SITDL simulation was run with fixed τ_Glu_ and single glutamate spike signal, and corresponding τ_Syn_ was then calculated. τ_Glu_ = 5 ms and 1410 ms correspond to τ_Syn_ of around 7 ms and 50 ms, respectively.

**
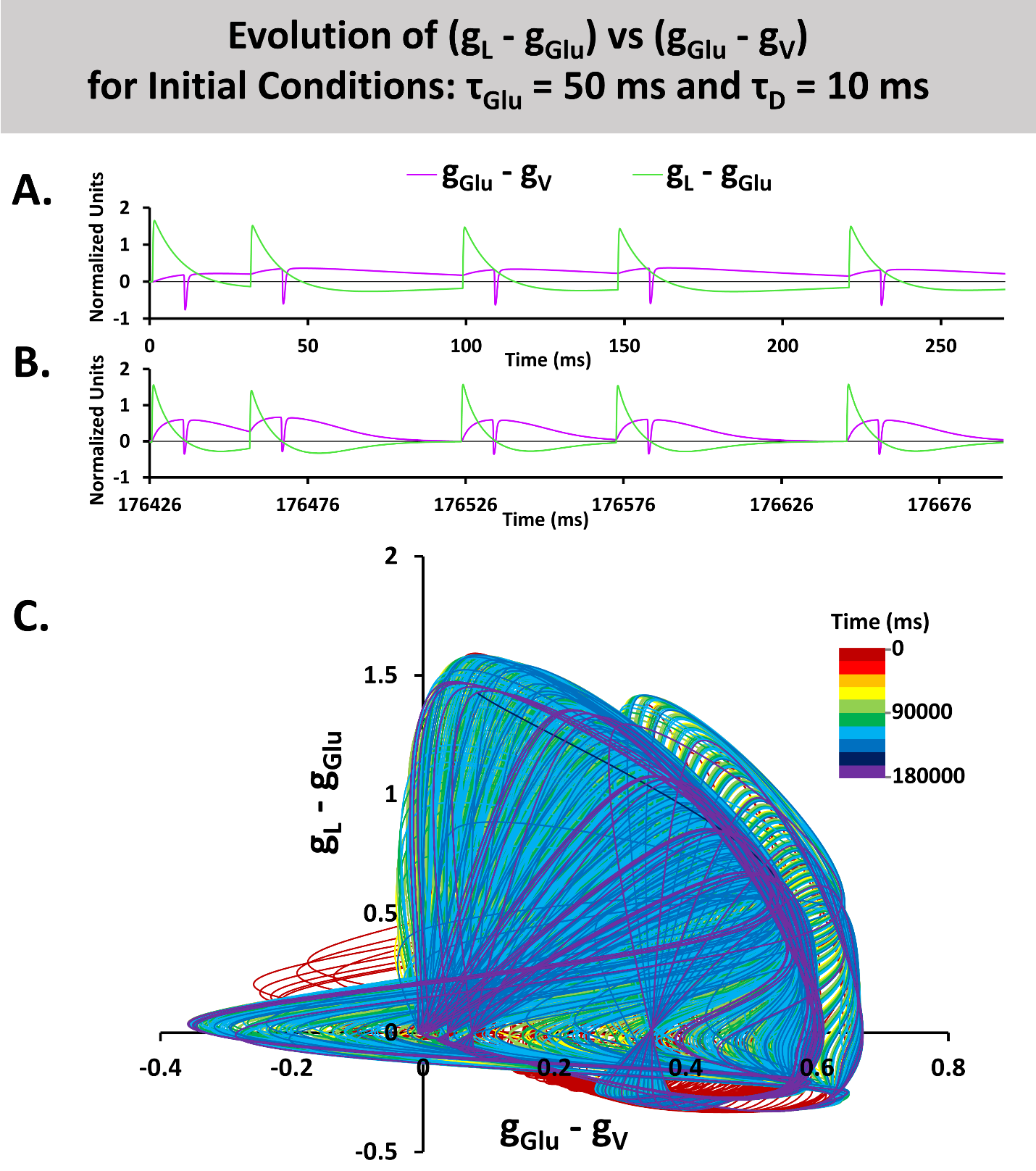
**

**Figure S4**. Evolution of (g_L_ - g_Glu_) vs (g_Glu_ - g_V_) for 180000 ms SITDL simulation with initial conditions of τ_Glu_ = 50 ms and τ_D_ = 10 ms. **A)** Changes in (g_Glu_ - g_V_) and (g_L_ - g_Glu_) for first 270 ms of the simulation. **B)** Changes in (g_Glu_ - g_V_) and (g_L_ - g_Glu_) for a 270 ms segment much later in the simulation. **C)** Phase portrait of (g_L_ - g_Glu_) vs (g_Glu_ - g_V_) values for the entire 180000 ms SITDL simulation, with time indicated by color. Notably, there is convergence to a stable trajectory after about 150000 ms simulation time (purple trajectory).


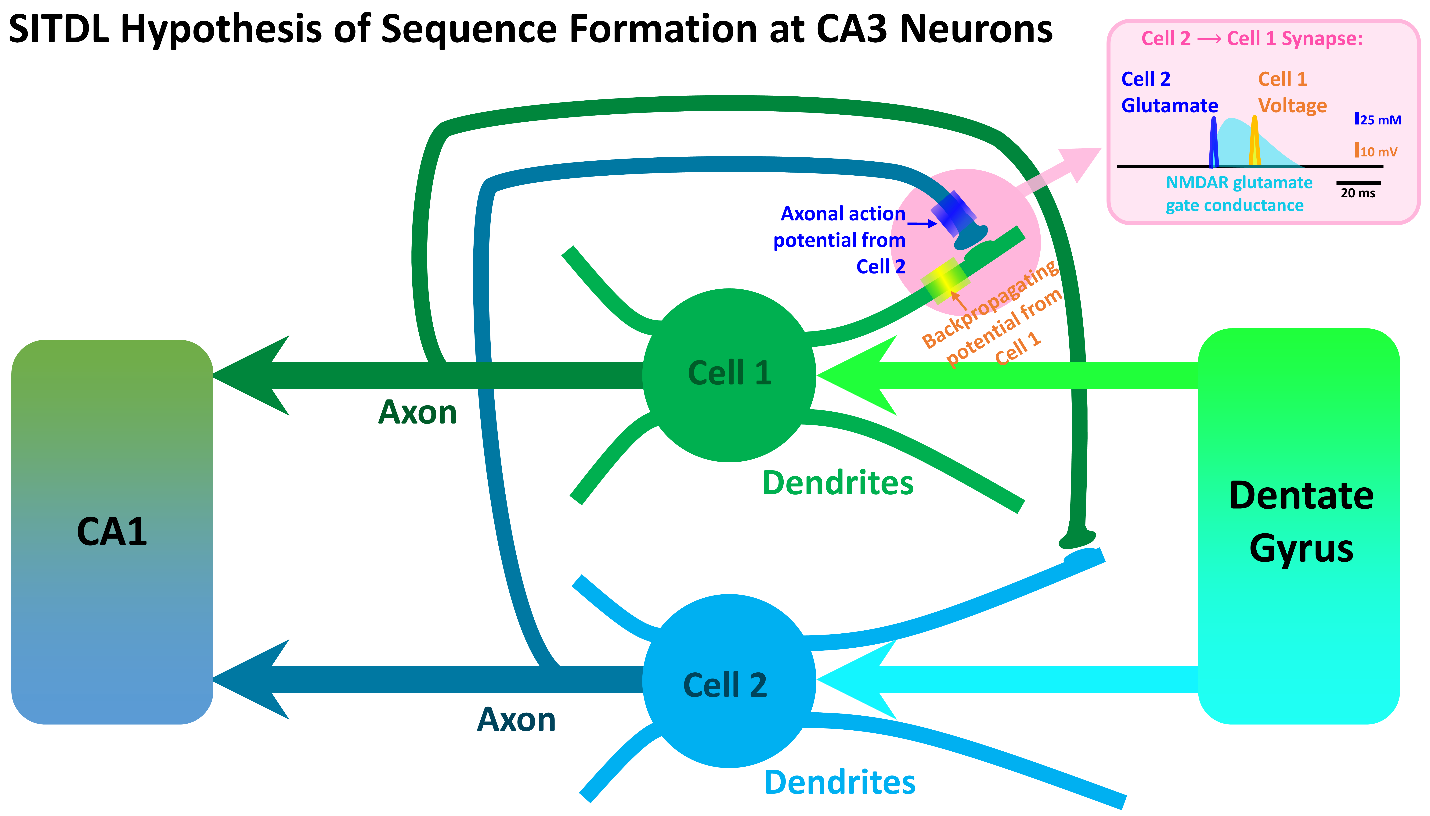


**Figure S5**. A hypothesis of how SITDL can enable memorization of sequences, as in place cell sequence formation, at CA3 neurons. Specifically, Cell 2 receives input from the dentate gyrus and fires, releasing glutamate onto Cell 1. In the meantime, Cell 1 also fires due to input from the dentate gyrus, and due to backpropagation of the potential along the dendrites of Cell 1, this results in NMDARs at the Cell 2 to Cell 1 synapse receiving a glutamate signal from Cell 2, followed by a backpropagating voltage signal from Cell 1. If SITDL mechanisms are in place, then the synapse may potentially learn the time difference between Cell 2 and Cell 1 activation. An entire CA3 network with these kinds of connections and mechanisms may therefore be capable of learning very complex sequences of activation.
